# Supplementary material for: The Extracellular Metabolome Stratifies Low and High Risk Potentially Premalignant Oral Keratinocytes and Identifies Citrate as a Potential Non-Invasive Marker of Tumour Progression
Source: Cancers (Basel). 2021 Aug 21;13(16):4212. doi: 10.3390/cancers13164212 (PMC8394991; doi:10.3390/cancers13164212)
Supplement: Supplementary file 1 [file cancers-13-04212-s001.zip › cancers-1349853-supplementary.pdf]

The extracellular metabolome stratifies low and high risk potentially premalignant oral keratinocytes and identifies citrate as a potential non-invasive marker of tumour progression

Table S1A. Molecular characteristics of the cell lines used in the study.

Cell Line	Site	Ploidy	P16^{INK4A}	P53	Telomerase	Notch Mutation	HPV16
NHOK810 Mortal	Buccal Mucosa	ND	+	+	ND	ND	ND
NHOK881 Mortal	Buccal Mucosa	ND	+	+	ND	ND	ND
D6 Mortal	Base of tongue	Diploid	+	+	-	ND	-
D25 Mortal	Floor of mouth	Diploid	+	+	-	ND	-
D30 Mortal	Floor of mouth	Diploid	+	+	-	ND	-
E4 Mortal	Lateral Tongue	Diploid	+	+	-	No	-
D17 Mortal (extended lifespan)	Buccal mucosa	Pseudodiploid *	-	+	-	Yes	-
D4 Immortal	Floor of mouth/ventral tongue	Aneuploid	-	-	+	No	-
D9 Immortal	Ventral Tongue	Aneuploid	-	-	+	No	-
D19 Immortal	Lateral Tongue	Aneuploid	-	-	+	No	-
D20 Immortal	Lateral Tongue	Aneuploid	-	-	+	No	-
D34 Immortal	Lateral Tongue	Aneuploid	-	-	+	No	-
D35 Immortal	Floor of mouth/ventral tongue	Aneuploid	-	-	+	Yes	-
DOK Immortal	Tongue	Aneuploid	-	-	+	ND	-

* Trisomy of chromosomes 2, 5, 7 and 9 (N. Thakker – personal communication); +/- = Heterozygous mutation.

Table S1B. Clinical and pathological characteristics of the donor tissues.

Cell Line	Site	Dysplasia Stage	Smoking	Age	Gender	Progression to SCC within 5 years	Meta-chronous SCC
NHOK 810	Buccal Mucosa	N/A	ND	ND	ND	N/A	N/A
NHOK 881	Buccal Mucosa	N/A	ND	ND	ND	N/A	N/A
D6	Base of tongue	Moderate/ severe	+	58	M	No	No
D25	Floor of mouth	Severe	+	55	M	No	No
D30	Floor of mouth	Mild	+	52	M	No	No
E4	Lateral Tongue	Carcinoma-in situ	+	65	F	Yes	No
D17	Buccal mucosa	Mild/ moderate	+	61	M	N/A	Yes
D4	Floor of mouth/ ventral tongue	Carcinoma-in situ	+	51	M	N/A	Yes
D9	Ventral Tongue	Mild/ moderate	-	84	M	No	No
D19	Lateral Tongue	Severe/ Carcinoma-in situ	+	53	M	Yes	No
D20	Lateral Tongue	Carcinoma-in situ	-	50	M	Yes	No
D34	Lateral Tongue	Moderate	-	54	F	No	No
D35	Floor of mouth/ ventral tongue	Severe/ Carcinoma-in situ	+	68	M	Yes	No
DOK	Tongue	Mild/ moderate	+	57	M	N/A	Yes

M = Male; F= Female; HPV = Human papilloma virus; N/A = Not applicable; ND = Not determined

Table S2A. Metabolites distinguishing normal oral keratinocyte lines NHOK810 (low senescence) and NHOK881 (high senescence) $p < 0.05$.

Sub pathway	Metabolite	Ratio NHOK881/NHOK 810	*p* Value (Welch's T test)
Leucine, isoleucine and valine metabolism	isovalerate	0.41	0.006
	3-hydroxyisobutyrate	0.42	0.004
	3-methyl-2-oxobutyrate	0.78	0.03
	beta-hydroxyisovalerate	0.37	0.004
Methionine, Cysteine, SAM and Taurine Metabolism	2-aminobutyrate	0.63	0.048
	cysteine	1.32	0.04
Gamma-glutamyl Amino Acid	gamma-glutamylglutamate	0.46	0.01
	gamma-glutamylleucine	0.83	0.01
	gamma-glutamylmethionine	0.66	0.009
Phenylalanine and Tyrosine Metabolism	3-(4-hydroxyphenyl)lactate	0.46	0.02
	4-hydroxyphenylpyruvate	1.6	0.04
Dipeptide	aspartylphenylalanine	0.38	0.002
Ketone bodies	3-hydroxybutyrate (BHBA)	0.77	0.007
Fatty Acid, Monohydroxy	3-hydroxydecanoate	1.78	0.04
Polyunsaturated fatty acid (n3 and n6)	linolenate [alpha or gamma; (18:3n3 or 6)]	2.2	0.05
	arachidonate (20:4n6)	1.48	0.03
Benzoate metabolism	hippurate	1.18	0.04
Lysolipid	2-oleoylglycerophosphocholine*	0.59	0.03
Fructose, Mannose and Galactose Metabolism	mannose	0.64	0.04
Pyrimidine metabolism thymine containing	thymine	13.5	0.002
	3-aminoisobutyrate	0.28	0.01
Pyrimidine metabolism uracil containing	pseudouridine	0.75	0.03
	uracil	2.43	0.0003
	3-ureidopropionate	0.08	0.008
Purine Metabolism, (Hypo)Xanthine/Inosine containing	xanthine	4.63	0.04

Values highlighted in red elevated values highlighted in green depleted.

Table S2B. Metabolites distinguishing normal oral keratinocyte lines NHOK810 (low senescence) and NHOK881 (high senescence) with the background subtracted and normalised for cell number $p < 0.05$ in bold.

Sub pathway	Metabolite	Ratio NHOK881/NHOK 810	P value (Welch's T test)
Leucine, isoleucine and valine metabolism	isovalerate	0.83	0.51
	3-hydroxyisobutyrate	1.01	0.96
	3-methyl-2-oxobutyrate	1.94	0.11
	beta-hydroxyisovalerate	0.97	0.90
Methionine, Cysteine, SAM and Taurine Metabolism	2-aminobutyrate	0.04	0.63
	cysteine	1.58	0.79
Gamma-glutamyl Amino Acid	gamma-glutamylglutamate	0.97	0.95
	gamma-glutamylleucine	1.53	0.06
	gamma-glutamylmethionine	1.28	0.44
Phenylalanine and Tyrosine Metabolism	3-(4-hydroxyphenyl)lactate	1.25	0.58
	4-hydroxyphenylpyruvate	4.79	0.06
Dipeptide	aspartylphenylalanine	5.43	0.06
Ketone bodies	3-hydroxybutyrate (BHBA)	1.88	0.11
Fatty Acid, Monohydroxy	**3-hydroxydecanoate**	**5.93**	**0.03**
Polyunsaturated fatty acid (n3 and n6)	linolenate [alpha or gamma; (18:3n3 or 6)]	1.5	0.50
	arachidonate (20:4n6)	2.66	0.30
Benzoate metabolism	hippurate	0.36	0.50
Lysolipid	2-oleoylglycerophosphocholine*	0.28	0.003
Fructose, Mannose and Galactose Metabolism	mannose	1.58	0.37
Pyrimidine metabolism thymine containing	**thymine**	**>44**	**0.02**
	**3-aminoisobutyrate**	**0**	**0.03**
Pyrimidine metabolism uracil containing	pseudouridine	1.85	0.18
	**uracil**	**8.99**	**0.049**
	**3-ureidopropionate**	**0.06**	**0.005**
Purine Metabolism, (Hypo)Xanthine/Inosine containing	xanthine	2.93	0.07

Values highlighted in red elevated values highlighted in green depleted.

Table S3A. Metabolites distinguishing LR MPPOLs from normal oral keratinocytes > 2 fold, $p < 0.05$ Q < 0.05.

	Metabolite	Ratio LR MPPOL/ Normal	P value (Welch's T test)	Q value
Glycine, serine and threonine metabolism	homoserine	0.49	0.0079	0.0110
Alanine and aspartate metabolism	asparagine	2.21	0.0029	0.0057
Lysine metabolism	2-aminoadipate	7.17	0.0034	0.0062
Phenyl alanine and tyrosine metabolism	3-(4-hydroxyphenyl)lactate	2.55	0.0033	0.0062
	phenol sulfate	2.26	0.0001	0.0005
	phenyllactate (PLA)	2.55	0.0008	0.0024
Tryptophan metabolism	anthranilate	3.45	0.0002	0.0008
	indolelactate	3.09	0.0006	0.0023
Leucine, isoleucine and valine metabolism	3-hydroxyisobutyrate	2.28	0.0063	0.0096
	3-hydroxy-2-ethylpropionate	8.08	<0.0001	0.0003
	isovalerate	2.52	0.0027	0.0054
	beta-hydroxyisovalerate	2.22	0.01	0.01
Urea cycle;argenine and proline metabolism	dimethylarginine (SDMA + ADMA)	3.10	0.0153	0.0167
Glutathione metabolism	cysteine-glutathione disulfide	0.27	0.0001	0.0006
Gamma-glutamyl amino acid	gamma-glutamyltyrosine	2.34	<0.0001	0.0001
	gamma-glutamylthreonine	2.58	0.0008	0.0024
	gamma-glutamylmethionine	2.06	0.0001	0.0007
Long chain fatty acid	10-heptadecenoate (17:1n7)	2.29	0.0017	0.0041
	myristoleate (14:1n5)	2.18	0.0057	0.0092
	palmitoleate (16:1n7)	2.25	0.0075	0.0106
Polyunsaturated fatty acid (n3 and n6)	dihomo-linolenate (20:3n3 or n6)*	2.71	0.0047	0.0082
	linolenate [alpha or gamma; (18:3n3 or 6)]	3.18	0.0008	0.0024
Sterol metabolism	7-alpha-hydroxy-3-oxo-4-cholestenoate (7-Hoca)	5.10	0.0001	0.0006
Steroid	cortisone	0.31	<0.0001	<0.0001
Phospholipid metabolism	ethanolamine	2.96	0.0003	0.0011
Eicosanoid	prostaglandin E1	12.76	0.0001	0.0005
	prostaglandin E2	4.73	0.006	0.0093
Ketone bodies	3-hydroxybutyrate (BHBA)	2.24	<0.0001	0.0001
Fatty acid monohydroxy	2-hydroxypalmitate	2.52	0.0001	0.0006
	4-hydroxybutyrate (GHB)	0.47	0.0026	0.0054
Glycerolipid metabolism	glycerol	0.39	0.0001	0.0006
Purine metabolism	urate	2.19	0.0209	0.0203
Pyrimidine metabolism uracil containing	3-ureidopropionate	4.62	0.0136	0.0156
	uracil	0.42	0.0158	0.0168
Pyrimidine metabolism uracil containing	thymine	0.02	0.0106	0.0138
Cofactors and vitamins	alpha-lipoate	0.23	<0.0001	0.0001

Values highlighted in red elevated values highlighted in green depleted.

Table S3B. Metabolites distinguishing LR MPPOLs from normal oral keratinocyte line NHOK 810 with the background subtracted and normalised for cell number > 2 fold, $p < 0.05$.

	Metabolite	Ratio LR MPPOL/Normal oral keratinocytes	P value (Welch's T test)
Glycine, serine and threonine metabolism	homoserine	0.18	0.005
Alanine and aspartate metabolism	asparagine	6.6	0.20
Phenyl alanine and tyrosine metabolism	phenol sulfate	13.5	0.005
	phenyllactate (PLA)	3.94	0.006
Tryptophan metabolism	anthranilate	5.24	0.0001
	indolelactate	3.27	0.002
Leucine, isoleucine and valine metabolism	3-hydroxy-2-ethylpropionate	18.35	0.0007
	isovalerate	2.44	0.003
	beta-hydroxyisovalerate	1.79	0.03
Glutathione metabolism	cysteine-glutathione disulfide	0.15	0.06
Gamma-glutamyl amino acid	gamma-glutamyltyrosine	1.84	0.01
	gamma-glutamylthreonine	2.60	0.02
	gamma-glutamylmethionine	2.10	0.0004
Lysolipid	1-stearoylglycerophosphoinositol	3.92	0.009
Long chain fatty acid	10-heptadecenoate (17:1n7)	3.24	7.34 x 10^{-5}
	myristoleate (14:1n5)	3.33	9.02 x10^{-5}
	palmitoleate (16:1n7)	3.88	0.006
	oleate	2.23	0.04
Polyunsaturated fatty acid (n3 and n6)	dihomo-linolenate (20:3n3 or n6)*	3.47	0.03
	dihomo-linoleate (20:2n6)	1.75	0.83
	linolenate [alpha or gamma; (18:3n3 or 6)]	5.08	0.003
	linoleate (18:2n6)	2.88	0.02
Sterol metabolism	7-alpha-hydroxy-3-oxo-4-cholestenoate (7-Hoca)*	4.52	0.0004
Phospholipid metabolism	ethanolamine	2.42	0.02
Eicosanoid	prostaglandin E1	12.55	0.01
	prostaglandin E2	19.69	0.003
Fatty acid monohydroxy	2-hydroxypalmitate**	3.13	0.0009
	3-hydroxydecanoate	2.29	0.02
	4-hydroxybutyrate (GHB)	0.41	0.0007
Glycerolipid metabolism	glycerol	0.33	0.0002
Pyrimidine metabolism uracil containing	3-ureidopropionate	2.49	0.008
Cofactors and vitamins	alpha-lipoate	0.24	0.0007

Values highlighted in red elevated values highlighted in green depleted.

Table S4A. Metabolites distinguishing HR IPPOLs from normal oral keratinocytes > 2 fold; p < 0.05 q < 0.05 in bold.

	Metabolite	HR IPPOL/Normal	P value (Welch's T test)	Q value (FDR)
Leucine, isoleucine and valine metabolism	**beta-hydroxyisovalerate**	**0.19**	< 0.0001	0.0007
Leucine, isoleucine and valine metabolism	**3-hydroxyisobutyrate**	**0.42**	0.0042	0.02
Methionine, cysteine, SAM and taurine metabolism	homocysteine	6.45	0.0447	0.1013
Methionine, cysteine, SAM and taurine metabolism	**cysteine**	**0.46**	0.0002	0.0037
Urea cycle;argenine and proline metabolism	dimethylarginine (SDMA + ADMA)**	2.93	0.0351	0.0881
Glutathione metabolism	glutathione, reduced (GSH)	23.81	0.0263	0.0745
Glutathione metabolism	**glutathione, oxidized (GSSG)**	**52.12**	0.0004	0.0047
TCA cycle	**Citrate**	**3.66**	0.0121	0.0443
Long chain fatty acid	**myristoleate (14:1n5)**	**0.34**	0.0008	0.007
Long chain fatty acid	**palmitoleate (16:1n7)**	**0.26**	0.0004	0.0043
Long chain fatty acid	**10-heptadecenoate (17:1n7)***	**0.40**	0.0009	0.0071
Polyunsaturated fatty acid (n3 and n6)	**docosapentaenoate (n3 DPA; 22:5n3)**	**0.38**	<0.0001	0.0006
Polyunsaturated fatty acid (n3 and n6)	**docosahexaenoate (DHA; 22:6n3)**	**0.39**	<0.0001	0.0004
Polyunsaturated fatty acid (n3 and n6)	**linoleate (18:2n6)****	**0.37**	0.0027	0.0165
Polyunsaturated fatty acid (n3 and n6)	**linolenate [alpha or gamma; (18:3n3 or 6)]**	**0.41**	0.0031	0.0176
Polyunsaturated fatty acid (n3 and n6)	**dihomo-linolenate (20:3n3 or n6)****	**0.33**	0.001	0.0073
Polyunsaturated fatty acid (n3 and n6)	**arachidonate (20:4n6)**	**0.40**	<0.0001	0.0004
Phospholipid metabolism	**glycerophosphorylcholine (GPC)**	0.47	<0.0001	0.0007
Purine metabolism	hypoxanthine	0.37	0.0491	0.1041
Purine metabolism	**N1-methyladenosine**	**2.11**	0.0129	0.0443
Pyrimidine metabolism	**uracil**	**0.49**	0.0133	0.0443

Values highlighted in red elevated values highlighted in green depleted.

Table S4B. Metabolites distinguishing HR IPPOLs from normal oral keratinocyte line NHOK810 with the background subtracted and normalised for cell number > 2 fold, $p < 0.05$ in bold.

	Metabolite	HR IPPOL/Normal	P value (Welch's T test)
Leucine, isoleucine and valine metabolism	**beta-hydroxyisovalerate**	**0.06**	**0.002**
	**3-hydroxyisobutyrate**	**0.17**	**0.009**
Methionine, cysteine, SAM and taurine metabolism	**homocysteine**	**>2.34**	**0.03**
	cysteine	0.79	0.14
Urea cycle;argenine and proline metabolism	dimethylarginine (SDMA + ADMA)**	2.04	0.11
Glutathione metabolism	glutathione, reduced (GSH)	ND*	0.08
	**glutathione, oxidized (GSSG)**	**ND***	**0.01**
TCA cycle	**citrate**	**2.5**	**0.007**
Long chain fatty acid	myristoleate (14:1n5)	0.21	0.08
	**palmitoleate (16:1n7)**	**0.26**	**0.004**
	**10-heptadecenoate (17:1n7)***	**0.32**	**0.03**
Polyunsaturated fatty acid (n3 and n6)	docosapentaenoate (n3 DPA; 22:5n3)	0.60	0.17
	docosahexaenoate (DHA; 22:6n3)	0.67	<0.20
	linoleate (18:2n6)**	0.86	0.36
	linolenate [alpha or gamma; (18:3n3 or 6)]	0.89	0.39
	dihomo-linolenate (20:3n3 or n6)**	0.62	0.11
	arachidonate (20:4n6)	1.2	0.43
Phospholipid metabolism	**glycerophosphorylcholine (GPC)**	**0.40**	**4.26×10^{-5}**
Purine metabolism	hypoxanthine	0.62	0.27
	N1-methyladenosine	1.26	0.35
Pyrimidine metaboliosm	uracil	1.1	0.85

Values highlighted in red elevated values highlighted in green depleted.* Undetectable in NHOK 810.

Table S5A. Metabolites distinguishing rapidly progressing HR IPPOLs from normal oral keratinocytes > 2 fold, $p < 0.05$ Q < 0.05 in bold.

Sub pathway	Metabolite	Rapidly progressing HR IPPOL/Normal	P value (Welch's T test)	Q value
Uridine metabolism	glutamate	0.47	0.0309	0.1471
Leucine, isoleucine and valine metabolism	**beta-hydroxyisovalerate**	**0.19**	0.0002	0.0167
	alpha-hydroxyisovalerate	0.50	0.0336	0.1513
	3-hydroxyisobutyrate	0.47	0.0187	0.1154
Urea cycle;argenine and pro-line metabolism	dimethylarginine (SDMA + ADMA)	3.57	0.0174	0.1117
Glutathione metabolism	glutathione, oxidized (GSSG)	26.77	0.0262	0.1367
TCA cycle	citrate	4.88	0.0334	0.1513
Long chain fatty acid	**myristoleate (14:1n5)**	**0.39**	0.0024	0.0378
	**palmitoleate (16:1n7)**	**0.32**	0.0022	0.0378
	**10-heptadecenoate (17:1n7)****	**0.42**	0.0019	0.0378
Polyunsaturated fatty acid (n3 and n6	**docosapentaenoate (n3 DPA; 22:5n3)**	**0.40**	0.0009	0.0372
	**docosahexaenoate (DHA; 22:6n3)**	**0.43**	0.0014	0.0378
	linoleate (18:2n6)	0.40	0.0054	0.0593
	linolenate [alpha or gamma; (18:3n3 or 6)]	0.41	0.0050	0.0593
	dihomo-linolenate (20:3n3 or n6)	0.38	0.0043	0.0553
	**arachidonate (20:4n6)**	**0.44**	0.0001	0.0167
Glycerolipid metabolism	**glycerol**	**0.47**	0.0024	0.0378
Purine metabolism	hypoxanthine	0.32	0.0271	0.1368
	N1-methyladenosine	2.27	0.0158	0.1052
Ascorbate and aldarate metab-olism	gulono-1,4-lactone	2.94	0.0220	0.1272

Values highlighted in red elevated in HR IPPOL keratinocytes values highlighted in green depleted.

Table S5B. Metabolites distinguishing rapidly progressing HR IPPOLs from normal oral keratinocyte line NHOK810 with the background subtracted and normalised for cell number > 2 fold, P < 0.05 in bold.

Sub pathway	Metabolite	Rapidly progressing HR IPPOL/Normal	P value (Welch's T test)
Uridine metabolism	**glutamate**	**0.45**	**0.009**
Leucine, isoleucine and valine metabolism	**beta-hydroxyisovalerate**	**0.05**	**0.001**
	**alpha-hydroxyisovalerate**	**0.23**	**0.002**
	**3-hydroxyisobutyrate**	**0.17**	**0.01**
Urea cycle;argenine and proline metabolism	dimethylarginine (SDMA + ADMA)	2.1	0.14
Glutathione metabolism	glutathione, oxidized (GSSG)	ND*	0.16
TCA cycle	**citrate**	**2.88**	**0.03**
Long chain fatty acid	myristoleate (14:1n5)	0.37	0.41
	**palmitoleate (16:1n7)**	**0.36**	**0.046**
	10-heptadecenoate (17:1n7)**	0.38	0.11
Polyunsaturated fatty acid (n3 and n6	docosapentaenoate (n3 DPA; 22:5n3)	0.63	0.43
	docosahexaenoate (DHA; 22:6n3)	0.73	0.49
	linoleate (18:2n6)	0.94	0.87
	linolenate [alpha or gamma; (18:3n3 or 6)]	0.96	0.91
	dihomo-linolenate (20:3n3 or n6)	0.67	0.42
	arachidonate (20:4n6)	0.87	0.76
Glycerolipid metabolism	glycerol	**0.21**	**$8.93x 10^{-5}$**
Purine metabolism	hypoxanthine	2.21	0.16
	N1-methyladenosine	1.18	0.56
Ascorbate and aldarate metabolism	gulono-1,4-lactone	**4.73**	**0.05**

Values highlighted in red elevated in HR IPPOL keratinocytes values highlighted in green depleted. Values highlighted in red elevated values highlighted in green depleted.* Undetectable in NHOK 810.

Table S6A. Metabolites distinguishing LR MPPOLs from HR IPPOL keratinocytes. > 2 fold, $p < 0.05$ Q < 0.05.

Sub pathway	Metabolite	HR IPPOL/ LR MPPOL	P value (Welch's T test)	Q value
Glycine serine and threonine metabolism	glycine	0.48	<0.0001	0.0001
Lysine metabolism	2-aminoadipate	0.16	0.0038	0.0046
Phenylalanine and tyrosine metabolism	phenyllactate	0.21	<0.0001	<0.0001
	3-(4-hydroxyphenyl)lactate	0.29	<0.0001	<0.0001
	phenol sulphate	0.49	<0.0001	0.0001
Tryptophan metabolism	indoleacetate	0.24	<0.0001	<0.0001
	kyneurenine	2.08	0.0245	0.0197
Leucine,isoleucine and valine metabolism	isovalerate	0.27	<0.0001	<0.0001
	beta-hydroxyisovalerate	0.08	<0.0001	<0.0001
	alpha-hydroxyisovalerate	0.39	0.0002	0.0004
	2-hydroxy-3-methylvalerate	0.44	0.0001	0.0003
	3-hydroxy-2-ethylpropionate	0.13	<0.0001	<0.0001
	3-hydroxyisobutyrate	0.18	<0.0001	<0.0001
Methionine, cysteine, SAM and taurine metabolism	cysteine	0.44	0.0003	0.0005
	homocysteine	7.14	0.0284	0.0219
Glutathione metabolism	glutathione, reduced (GSH)	25.00	0.0263	0.0207
	glutathione, oxidized (GSSG)	50.00	0.0004	0.0007
	cysteine glutathione disulphide	4.35	0.0001	0.0003
Glycolysis, gluconeogenesis and pyruvate metabolism	pyruvate	2.04	<0.0001	0.0001
TCA cycle	citrate	3.03	0.0521	0.0374
Long chain fatty acid	myristoleate (14:1n5)	0.15	<0.0001	<0.0001
	palmitoleate (16:1n7)	0.11	0.0114	<0.0001
	10-heptadecenoate (17:1n7)	0.17	<0.0001	<0.0001
	oleate (18:1n9)	0.43	0.0001	0.0002

Values highlighted in red elevated values in LR MPPOL keratinocytes highlighted in green depleted.

Table S6B. Metabolites distinguishing LR MPPOLs from HR IPPOL keratinocytes with the background subtracted and normalised for cell number $p < 0.05$ in bold.

Sub pathway	Metabolite	HR IPPOL/ LR MPPOL	P value (Welch's T test)
Glycine serine and threonine metabolism	**glycine**	**0.22**	**0.0001**
Lysine metabolism	**2-aminoadipate**	**0.04**	**3.22×10^{-5}**
Phenylalanine and tyrosine metabolism	**phenyllactate**	**0.07**	**1.56×10^{-7}**
	**3-(4-hydroxyphenyl)lactate**	**0.19**	**7.25×10^{-10}**
	**phenol sulphate**	**0.09**	**1.49×10^{-6}**
Tryptophan metabolism	**indoleacetate**	**0.08**	**1.81×10^{-9}**
	kyneurenine	14.5	0.07
Leucine,isoleucine and valine metabolism	**isovalerate**	**0.14**	**9.85×10^{-9}**
	**beta-hydroxyisovalerate**	**0.04**	**7.01×10^{-9}**
	**alpha-hydroxyisovalerate**	**0.25**	**1.31×10^{-5}**
	**2-hydroxy-3-methylvalerate**	**0.24**	**1.00×10^{-5}**
	**3-hydroxy-2-ethylpropionate**	**0.02**	**3.24×10^{-10}**
	**3-hydroxyisobutyrate**	**0.10**	**1.76×10^{-10}**
Methionine, cysteine, SAM and taurine metabolism	cysteine	0.32	0.18
	homocysteine	20.4	0.16
Glutathione metabolism	glutathione, reduced (GSH)	ND*	0.27
	glutathione, oxidized (GSSG)	ND*	0.11
	cysteine glutathione disulphide	4.35	0.07
Glycolysis, gluconeogenesis and pyruvate metabolism	pyruvate	1.89	0.11
TCA cycle	citrate	1.72	0.21
Long chain fatty acid	**myristoleate (14:1n5)**	**0.22**	**7.52×10^{-9}**
	**palmitoleate (16:1n7)**	**0.22**	**2.80×10^{-9}**
	**10-heptadecenoate (17:1n7)**	**0.32**	**9.24×10^{-10}**
	**oleate (18:1n9)**	**0.53**	**0.02**

Values highlighted in red elevated values in HR IPPOL keratinocytes highlighted in green depleted. Undetectable in LR MPPOL.

Table S7A. Metabolites distinguishing HR IPPOL keratinocytes from LR MPPOL and normal oral keratinocyte NHOK810 > 2 fold, $p < 0.05$ Q < 0.05 in bold.

Sub pathway	Metabolite	HR IPPOL/ LR MPPOL and normal keratinocyte NHOK810	P value (Welch's T test)	Q value
Phenylalanine and tyrosine metabolism	**phenyllactate**	0.36	0.005	0.02
Tryptophan metabolism	**indoleacetate**	0.43	0.03	0.07
Leucine, isoleucine and valine metabolism	**isovalerate**	0.27	0.001	0.009
	**beta-hydroxyisovalerate**	0.14	< 0.0001	0.0002
	3-hydroxy-2-ethylpropionate	0.27	0.03	0.07
	**3-hydroxyisobutyrate**	0.31	0.003	0.02
Methionine, cysteine, SAM and taurine metabolism	**cysteine**	0.50	0.001	0.008
Glutathione metabolism	glutathione, reduced (GSH)	23.81	0.03	0.07
	**glutathione, oxidized (GSSG)**	43.05	0.0006	0.006
TCA cycle	citrate	3.11	0.03	0.07
Medium chain fatty acid	**caprylate**	0.58	0.0007	0.006
Long chain fatty acid	**myristoleate (14:1n5)**	0.24	< 0.0001	< 0.0001
	**palmitoleate (16:1n7)**	0.19	< 0.0001	0.0001
	**10-heptadecenoate (17:1n7)**	0.28	< 0.0001	0.0001
Polyunsaturated Fatty Acid (n3 and n6)	**docosapentaenoate (n3 DPA; 22:5n3)**	0.35	< 0.0001	0.0001
	**docosahexaenoate (DHA; 22:6n3)**	0.39	< 0.0001	0.0002
	**linoleate (18:2n6)**	0.30	< 0.0001	< 0.0001
	**linolenate [alpha or gamma; (18:3n3 or 6)]**	0.22	< 0.0001	0.0001
	**dihomo-linolenate (20:3n3 or n6)**	0.21	< 0.0001	0.0004
	**arachidonate (20:4n6)**	0.36	< 0.0001	0.0001
	**dihomo-linoleate (20:2n6)**	0.56	0.009	0.03
Ketone bodies	**3-hydroxybutyrate (BHBA)**	0.46	0.004	0.02
Eicosenoid	prostaglandin E2	0.45	0.03	0.07
	prostaglandin E1	0.19	0.02	0.07
Phospholipid	ethanolamine	0.42	0.02	0.07
Sterol	**7-alpha-hydroxy-3-oxo-4-cholestenoate (7-Hoca)**	0.29	0.005	0.02
Purine metabolism(hypo)xanthine/inosine containing	**hypoxthanthine**	0.40	0.002	0.01

Values highlighted in red elevated values in HR IPPOL relative to LR MPPOL and normal oral keratinocyte. Metabolites highlighted in green realtively depleted. .

Table S7B. Metabolites distinguishing HR IPPOL keratinocytes from LR MPPOL and normal oral keratinocyte line NHOK810 with the background subtracted and normalised for cell number $p < 0.05$ in bold.

Sub pathway	Metabolite	HR IPPOL /LR MPPOL and normal NHOK 810	P value (Welch's T test)
Phenylalanine and tyrosine metabolism	**phenyllactate**	**0.29**	**0.0008**
Tryptophan metabolism	**indoleacetate**	**0.10**	**0.001**
Leucine,isoleucine and valine metabolism	**isovalerate**	**0.16**	**0.004**
	**beta-hydroxyisovalerate**	**0.04**	**0.0004**
	**3-hydroxy-2-ethylpropionate**	**0.03**	**0.005**
	**3-hydroxyisobutyrate**	**0.11**	**0.0001**
Methionine, cysteine, SAM and taurine metabolism	cysteine	0.39	0.07
Glutathione metabolism	glutathione, reduced (GSH)	>2.54	0.08
	**glutathione, oxidized (GSSG)**	**>1.36**	**0.01**
TCA cycle	**citrate**	**1.83**	**0.03**
Medium chain fatty acid	**caprylate**	**0.49**	**0.04**
Long chain fatty acid	**myristoleate (14:1n5)**	**0.23**	**0.001**
	**palmitoleate (16:1n7)**	**0.28**	**0.002**
	**10-heptadecenoate (17:1n7)**	**0.32**	**0.001**
Polyunsaturated Fatty Acid (n3 and n6)	**docosapentaenoate (n3 DPA; 22:5n3)**	**0.38**	**0.04**
	docosahexaenoate (DHA; 22:6n3)	0.61	0.07
	**linoleate (18:2n6)**	**0.45**	**0.02**
	**linolenate [alpha or gamma; (18:3n3 or 6)]**	**0.64**	**0.009**
	**dihomo-linolenate (20:3n3 or n6)**	**0.41**	**0.008**
	arachidonate (20:4n6)	0.71	0.19
	**dihomo-linoleate (20:2n6)**	**0.75**	**0.02**
Ketone bodies	**3-hydroxybutyrate (BHBA)**	**0.12**	**0.0009**
Eicosenoid	prostaglandin E2	0.54	0.36
	**prostaglandin E1**	**0.001**	**0.03**
Phospholipid	**ethanolamine**	**0.09**	**0.003**
Sterol	**7-alpha-hydroxy-3-oxo-4-cholestenoate (7-Hoca)**	**0.07**	**0.005**
Purine metabolism(hypo)xanthine/inosine containing	hypoxthanthine	1.17	0.82

Values highlighted in red elevated values in HR IPPOL relative to LR MPPOL and normal oral keratinocyte. Metabolites highlighted in green relatively depleted.

Table S8A. Volatile metabolites distinguishing normal NHOK 810, LR MPPOL and HR IPPOL.

Biochemical	Pathway	Enthalpy of Vaporization	LR MPPOL v NHOK	HR IPPOL v LR MPPOL	HR IPPOL v LR MPPOL and NHOK
2-hydroxy-3-methylvalerate	Leucine, Isoleucine and Valine Metabolism	56.8±6.0	0.43	0.006	0.004
**2-hydroxybutyrate (AHB)**	**Methionine, Cysteine, SAM and Taurine Metabolism**	**55.2±6.0**	**0.04**	**0.005**	**0.004**
**3-hydroxybutyrate (BHBA)**	**Ketone Bodies**	**58.9±6.0**	**0.02**	**0.0005**	**0.0001**
**3-hydroxyisobutyrate**	**Leucine, Isoleucine and Valine Metabolism**	**57.0±6.0**	**0.02**	**0.0005**	**0.00006**
3-methyl-2-oxobutyrate	Leucine, Isoleucine and Valine Metabolism	44.8±6.0	0.12	0.007	0.004
3-methyl-2-oxovalerate	Leucine, Isoleucine and Valine Metabolism	47.0+/-6.0	0.43	0.03	0.02
**4-methyl-2-oxopentanoate**	**Leucine, Isoleucine and Valine Metabolism**	**47.0+/- 6.0**	**0.02**	**0.005**	**0.006**
alpha-hydroxyisovalerate	Leucine, Isoleucine and Valine Metabolism	55.1±6.0	0.43	0.003	0.001
beta-hydroxyisovalerate	Leucine, Isoleucine and Valine Metabolism	ND	0.12	0.0005	0.00006
**isovalerate**	**Leucine, Isoleucine and Valine Metabolism**	**45.4±6.0**	**0.02**	**0.0005**	**0.0002**

Table S8B. Metabolites distinguishing normal NHOK 810, LR MPPOL and HR IPPOL keratinocytes with the potential to be converted into volatile metabolites by oral bacteria.

Biochemical	Pathway	Enthalpy of Vaporization	LR MPPOL v NHOK	HR IPPOL v LR MPPOL	HR IPPOL v LR MPPOL and NHOK
**lysine**	**Lysine Metabolism**	NA	**0.02**	**0.001**	**0.005**
**methionine**	**Methionine Metabolism**	NA	**0.02**	**0.008**	**0.03**
**tryptophan**	**Tryptophan Metabolism**	NA	**0.02**	**0.02**	**0.04**

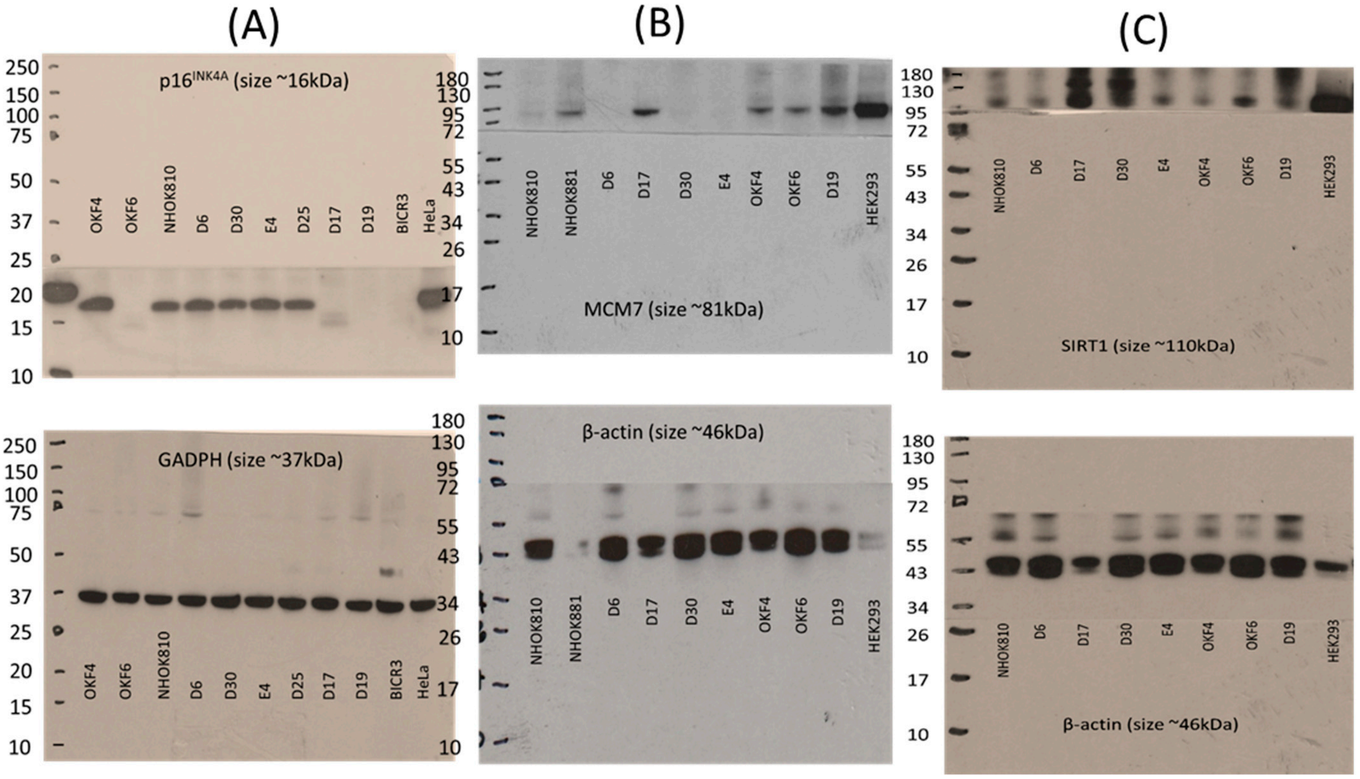

Figure S1. The whole Western blots are shown. Ladder used for (A) was Precision Plus Protein Standards Dual Color (Cat no. 161-0374, BioRad, Hertfordshire, UK) and (B) & (C) was Thermo Scientific PageRuler Prestained Protein Ladder (Cat no. 26617, ThermoScientific, Watham, USA).
